# Insulin-Like Growth Factor 1 Receptor (IGF1R) Expression and Survival in Operable Squamous-Cell Laryngeal Cancer

**DOI:** 10.1371/journal.pone.0054048

**Published:** 2013-01-24

**Authors:** Giannis Mountzios, Ioannis Kostopoulos, Vassiliki Kotoula, Ioanna Sfakianaki, Elena Fountzilas, Konstantinos Markou, Ilias Karasmanis, Sofia Leva, Nikolaos Angouridakis, Konstantinos Vlachtsis, Angelos Nikolaou, Ioannis Konstantinidis, George Fountzilas

**Affiliations:** 1 Department of Medical Oncology, 251 Airforce General Hospital, Athens, Greece; 2 Department of Pathology, Aristotle University of Thessaloniki School of Medicine, Thessaloniki, Greece; 3 Laboratory of Molecular Oncology, Hellenic Foundation for Cancer Research, Aristotle University of Thessaloniki School of Medicine, Thessaloniki, Greece; 4 Department of Medical Oncology, Papageorgiou Hospital, Aristotle University of Thessaloniki School of Medicine, Thessaloniki, Greece; 5 First Department of Otorhinolaryngology, AHEPA Hospital, Aristotle University of Thessaloniki School of Medicine, Thessaloniki, Greece; 6 ENT Department, G. Papanikolaou General Hospital, Thessaloniki, Greece; 7 ENT Department, Papageorgiou Hospital, Thessaloniki, Greece; Hemocentro de Ribeirão Preto, HC-FMRP-USP, Brazil

## Abstract

**Introduction:**

Prognosis of patients with operable laryngeal cancer is highly variable and therefore potent prognostic biomarkers are warranted. The insulin-like growth factor receptor (IGFR) signaling pathway plays a critical role in laryngeal carcinogenesis and progression.

**Patients and Methods:**

We identified all patients with localized TNM stage I–III laryngeal cancer managed with potentially curative surgery between 1985 and 2008. Immunohistochemical (IHC) expression of IGF1R-alpha, IGF1R-beta and IGF2R was evaluated using the immunoreactive score (IRS) and mRNA levels of important effectors of the IGFR pathway were assessed, including IGF1R, IGF-binding protein 3 (IGFBP3), suppressor of cytokine signaling 2 (SOCS2) and members of the MAP-kinase (MAP2K1, MAPK9) and phosphatidyl-inositol-3 kinase (PIK3CA, PIK3R1) families. Cox-regression models were applied to assess the predictive value of biomarkers on disease-free survival (DFS) and overall survival (OS).

**Results:**

Among 289 eligible patients, 95.2% were current or ex smokers, 75.4% were alcohol abusers, 15.6% had node-positive disease and 32.2% had received post-operative irradiation. After a median follow-up of 74.5 months, median DFS was 94.5 months and median OS was 106.3 months. Using the median IRS as the pre-defined cut-off, patients whose tumors had increased IGF1R-alpha cytoplasm or membrane expression experienced marginally shorter DFS and significantly shorter OS compared to those whose tumors had low IGF1R-alpha expression (91.1 vs 106.2 months, p = 0.0538 and 100.3 vs 118.6 months, p = 0.0157, respectively). Increased mRNA levels of MAPK9 were associated with prolonged DFS (p = 0.0655) and OS (p = 0.0344). In multivariate analysis, IGF1R-alpha overexpression was associated with a 46.6% increase in the probability for relapse (p = 0.0374). Independent predictors for poor OS included node-positive disease (HR = 2.569, p<0.0001), subglottic/transglottic localization (HR = 1.756, p = 0.0438) and IGF1R-alpha protein overexpression (HR = 1.475, p = 0.0504).

**Conclusion:**

IGF1R-alpha protein overexpression may serve as an independent predictor of relapse and survival in operable laryngeal cancer. Prospective evaluation of the IGF1R-alpha prognostic utility is warranted.

## Introduction

Laryngeal cancer represents the most common form of squamous-cell carcinoma of the head and neck (SCCHN) in populations with high prevalence of smoking. Accounting for approximately 160,000 new cases per year and for 2.5% of all tumors in males, laryngeal cancer remains a major disease burden worldwide [1]. Despite recent advances in the multidisciplinary management of the early stages of the disease, including surgical extirpation or larynx-preservation protocols implementing chemoradiotherapy, a substantial proportion of patients with localized or locally advanced disease will eventually relapse and die [2]. Even for patients who undergo the mutilating process of total laryngectomy, prognosis is highly variable and unpredictible, rendering the need to identify potent prognostic markers a medical priority. Accurate and reproducible estimation of prognosis in patients with early laryngeal cancer would enable aggressive treatment of those at high risk for relapse and would obviate potentially hazardous overtreatment in the group with a presumed favorable outcome.

The insulin and insulin-like growth factor receptor (IGFR) - mediated molecular pathways have recently emerged as important effectors of neoplastic transformation in various malignancies of the aerodigestive tract, including non small-cell lung cancer [3], [4], thyroid cancer [5], [6], esophageal cancer [7] and SCCHN [8]. The IGFR pathway comprises two ligands (IGF1 and IGF2), their binding proteins (the most abundant being IGFBP-3) and two receptors (IGF1R and IGF2R), [2]. Unlike insulin, which acts as a typical hormone, IGF1 and IGF2 have credentials both as circulating hormones with autocrine properties and as tissue growth factors; IGF1R has the capability of signal transduction through intracellular tyrosine kinase linked to RAS/RAF/mitogen activated protein kinase (MAPK) and the phosphatidyl-inositol-3 kinase (PI3K)-Akt pathways [9]. Precursor polypeptide cleavage leads to the presence of two IGF1R isoforms: Isoform alpha (IGF1R-alpha), which is preferentially expressed in many cancers and is able to bind to insulin, IGF1 and IGF2, and isoform-beta (IGF1R-beta), which binds exclusively to insulin [10]. IGF2R, on the other hand, binds only to IGF2, is structurally distinct in the sense that it lacks an intracellular tyrosine kinase domain, and is thus deprived from the ability to transduce mitogenic signals, acting mainly as a “buffer’ for IGF2 bioactivity [10]. The suppressor of cytokine signaling (SOCS) family of proteins are inhibitors of signaling pathways through a negative feedback loop involving mainly the inhibition of janus-kinase activity [11], [12]. More recent data, however, suggest that SOCS proteins and especially SOCS2 may also modulate IGF1R-mediated signaling [13], [14]. Importantly, preclinical and translational studies show that components of the IGFR-mediated pathway are implicated in SCCHN risk [15], angiogenesis [16], pharmacogenetics [17], [18], chemosensitivity [19], immunotherapy [20] and radiation response [21].

In a recent seminal work in the field [22] a multigene predictor of recurrence in early laryngeal cancer was developed and validated. In that study, a panel of genes coding for members of the IGFR pathway emerged as a potent prognostic tool able to discriminate patients with poor and favorable prognosis (p<0.0001 in the training set and p = 0.0001 in the first validation set) [22]. These results generated the hypothesis that protein expression and mRNA levels of the most important effectors of the IGFR pathway may be associated with clinical outcome in patients with early laryngeal cancer and may thus serve as predictive biological markers of relapse and survival in this setting. To test this hypothesis, we retrospectively examined tumoral gene transcription of important elements of the IGFR pathway and we evaluated protein expression of the same molecules in tumor cells from patients with surgically resected laryngeal cancer.

## Patient Characteristics and Methods

### Patient cohort

The study was conducted retrospectively in a cohort of patients with localized stage I–III laryngeal cancer (TNM 6th classification, available at: http://www.uicc.org/node/7735) managed between May 1985 and June 2008 at the ENT Department of “AHEPA” Hospital in Thessaloniki, Greece, with potentially curative resection with or without external beam irradiation. The present study was approved by the Bioethics Committee of the Aristotle University of Thessaloniki, School of Medicine. Waiver of consent was obtained from this committee for all patients included in the study before 2003. All patients included in the study after 2003 provided their informed written consent for the provision of biological material for future research studies. The study complied with the REMARK recommendations for tumor marker prognostic studies using biological material (available at http://www.ncbi.nlm.nih.gov/pmc/articles/PMC2361579).

Patients were clinically evaluated at regular monthly visits for the first year after surgery and every two months thereafter and staged with chest X-ray, cervical, chest and upper abdominal computed tomographic scans every six months for the first two years after surgery and yearly thereafter or sooner if clinically indicated. Other diagnostic or staging procedures were performed upon clinical indications or symptom alert. Treatment effect evaluation was performed according to the RECIST criteria for solid tumors (www.eortc.be/recist/documents/RECISTGuidelines.pdf).

### Histological Evaluation

**Figure 1:**
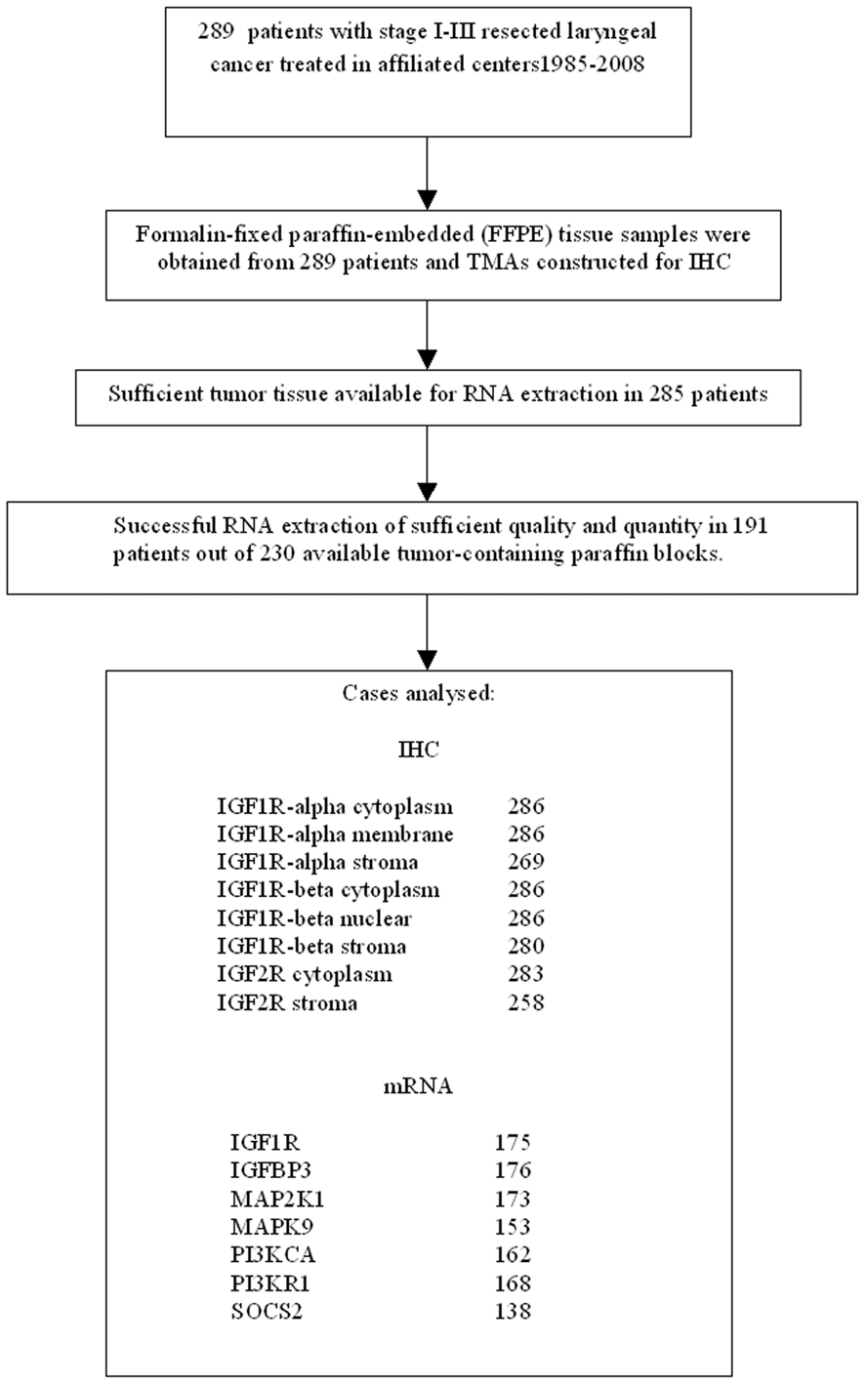
REMARK Flow Chart.

An experienced pathologist reviewed haematoxylin–eosin (H&E) stained slides from tissue blocks for adequacy of material and calculation of the percentage of tumor cells in each case. Two hundred eighty-nine formalin-fixed paraffin-embedded tissue blocks were histologically evaluated for tumor type and 285 of them with sufficient tumor tissue were marked for manual dissection. The latter was performed on routinely deparaffinized sections in order to increase tumor cell content in the extracted molecular templates, which contained >50% tumor cells in 64.2% of the cases and 30–50% in the remaining ones. The flow chart of the study including the corresponding sample numbers is presented in Figure 1 (REMARK diagram).

### RNA extraction and RT-qPCR methodology

A total of 230 tumor-containing paraffin blocks were available for molecular investigations (Figure 1). Macrodissection was performed in cases with <50% tumor cells in order to increase tumor cell content in molecular samples. Upon deparaffinization and overnight tissue fragment lysis with proteinase K at 56°C, RNA was extracted with TRIzol® LS Reagent and reverse transcribed with random primers (cat. no 48190-011) and the SuperScript® III Reverse Transcriptase (all reagents from Invitrogen/Life Technologies, Paisley, UK), according to the manufacturer's instructions. Upon UV measurements, 0.5–3 ug total RNA were reverse transcribed, cDNAs were normalized at 25 ng template/ul and kept at −20°C until use. Relative mRNA expression was assessed with qPCR and hydrolysis probes by using premade single tube TaqMan® Gene Expression Assays (Applied Biosystems/Life Technologies) in an ABI7900HT system under default conditions for the following targets (in parentheses: assay ID; Genbank reference; amplicon location; size): IGF1R (Hs00609566_m1; NM_000875.3; exons 10–11; 64 bp), IGFBP3 (Hs00426289_m1; NM_000598.4, NM_001013398.1; exons 4–5; 84 bp), MAP2K1 (Hs00983247_g1; NM_002755.3; exons 10–11; 68 bp), MAPK9 (Hs00177102_m1; NM_001135044.1, NM_002752.4, NM_139068.2, NM_139069.2, NM_139070.2; exons 2–3; 102 bp), PIK3CA (Hs00180679_m1; M_006218.2; exons 6–7; 104 bp), PIK3R1 (Hs00933163_m1; NM_001242466.1, NM_181504.3, NM_181523.2, NM_181524.1; exons 8–9, 9–10, 15–16; 82 bp), and, SOCS2 (Hs00919620_m1; NM_003877.3; exons 2–3; 91 bp). Samples were tested in 10 ul reactions (50 ng template/reaction) with TaqMan® Universal PCR Master Mix and were run in duplicates in 384-well plates. A commercially available reference RNA (TaqMan® Control Total RNA, cat. no 4307281, Applied Biosystems/Life Technologies) was used as a positive control in each run. As an endogenous control and for the normalization of Cq (quantification cycle, which is synonymous to CT [cycle threshold]) values, an assay targeting GUSB mRNA (beta-glucuronidase [#4333767F]) was used. GUSB was preferred over usually applied endogenous controls because (a) no pseudogenes have as yet been reported for this gene, and (b) it has been identified as one among the best preserved mRNA targets in FFPE tissues [23]. Relative quantification (RQ) was assessed in a linear mode as (40 – dCT) [24], whereby dCT = (avg CT target) – (avg CT GUSB). PCR assay stability was evaluated among runs with the reference RNA (inter-run deltaRQs: IGF1R 0.78; IGFBP3 0.72; MAP2K1 0.39; MAPK9 0.92; PIK3CA 0.62; PIK3R1 0.59; SOCS2 0.92). Exclusion criteria for sample RQ analysis were GUSB CT values higher than 36 for each duplicate; and, deltaRQ values higher than 0.85 per duplicate pair. With the above criteria, informative results were obtained in 191 (83%) out of 230 FFPE RNA samples. However, the number of informative samples ranged between 175 (75%) for IGF1R to 138 (59%) for SOCS2 resulting in only 118 (51%) informative samples for all targets. Thus, clustering of RQ values for the assessment of the corresponding gene expression profiles could not be assessed.

### TMA construction, immunohistochemistry and the scoring system

TMA construction was performed as previously described [25]. Briefly, serial 3 mm-thick sections form the original blocks or the TMA blocks, mounted on adhesion microscope slides, were cut at the Laboratory of Molecular Oncology of the Hellenic Foundation of Cancer Research, Aristotle University of Thessaloniki School of Medicine. The immunohistochemical (IHC) labeling was performed, using Bond Max™ (Leica Microsystems, Wezlar, Germany) and i6000 (Biogenex, San Ramon, CA) autostainers. The sections were stained with anti-IGF1R-alpha (clone 24–31, Lab Vision, Fremont, CA, at 1∶50 dilution for 1 h), anti- IGF1R-beta (C-20, sc-713, polyclonal antibody, raised against a peptide mapping at the C-terminus of IGF-Iâ molecule, Santa Cruz, Santa Cruz, CA, at 1∶250 dilution for 1 h) and IGF-2R (C-15,sc-14410, goat polyclonal antibody, Santa Cruz, at 1∶250 dilution for 1 h). The antigen-antibody complex was visualized using diaminobenzidine (DAB) as a chromogen. Slides were counterstained with Mayer's hematoxylin, washed in fresh water, dehydrated, and mounted. As external controls, we used cores (12 in total for each TMA) from various non-neoplastic and neoplastic tissues, including placenda, endometrium, kidney, tonsil, mammary gland, lymph node, prostate cancer and squamous-cell carcinoma of the head and neck. As internal controls, we used the neighboring epithelium of mucosal glands and the normal, hyperplastic and dysplastic columnar epithelium.

For the evaluation of IGF1R-alpha, IGF-1R-beta and IGF2R we used a semiquantitative approach based on staining intensity (SI) and percentage of positive cells (PP), to create the immunoreactive score (IRS) as follows: IRS = SIxPP, for each sample, as previously described [26], [27]. Intensity was scored as follows: 0 = no staining, 1 = weakly positive, 2 = moderately positive, and 3 = strongly positive. The scoring of the staining pattern was based on the percentage of positive tumor cells: 0 = 0–5%, 1 = 6–25%, 2 = 26–50%, 3 = 51–100%). The IRS score thus ranged from 0 to 9. The localization of staining for each protein was also indicated either as cytoplasmic or cytoplasmic/membranous or membranous.

All discordant cases were resolved within consensus meetings. In order to avoid false-positive findings arising from multiple cut-off calculations, we used the median value of the IRS as the pre-defined cut-off for each marker, as previously suggested [25]. A tumor sample was considered “high-expression” if the IRS was above or equal to the median and “low-expression” otherwise.

### Statistical Considerations

Regarding IHC expression of IGF1R and IGF2R the median IRS was pre-defined as the candidate cut-off for assessing the prognostic role of IGFR on disease-free survival (DFS) and overall survival (OS). Regarding biomarkers whose expression was measured on the mRNA level their distribution was examined for natural cut-offs. In case of no identification of a natural cut-off, the first, second and third quartiles were examined by means of univariate Cox regression analysis as possible thresholds. If a cut-off showed prognostic significance it was used to dichotomize the samples into low and high expressing tumors. Additional candidate cut-offs were explored with the use of receiver operating characteristic curves (ROC) using the 3-year DFS observed rate as binary outcome validated with bootstrap analysis. In the case that no cut-off was identified the median was used. In multivariate analysis significance was determined at the level of 15% and in univariate at 5% (two-sided).

DFS was measured from the time of diagnosis until verified disease progression, death or last contact and OS from diagnosis until death from any cause or date of last contact. Time-to-event distributions were estimated using Kaplan-Meier curves. Associations between biomarkers and with basic patient and tumor characteristics were examined using the Fisher's exact test for categorical variables and the Mann-Whitney or the Kruskall-Wallis test where appropriate for continuous variables. For continuous mRNA expression the correlations were calculated using the Pearson's correlation test. The statistical analysis complied with the reporting recommendations for tumor marker prognostic studies [28].

## Results

### Clinicopathological characteristics and outcome

Two hundred and eighty-nine patients, mostly males (95.8%), current or ex smokers (95.2%) and alcohol abusers (75.4%), with a median age of 63 years at diagnosis, were included in the present analysis. As shown in Table 1, diagnostic work-up led to diagnosis of squamous cell carcinoma of the larynx predominantly supraglottic (46.7%) or glottic (43.3% of cases), mostly stage T3–4 (77.8% of cases) and more often node-negative at clinical/radiological examination (84.1%). Initial management consisted of surgical resection of the tumor by means of total laryngectomy (84.1%) or by more conservative procedures (15.9%). A neck dissection was performed in 29.1% of cases, while post-operative external beam radiotherapy was administered in 32.2%.

**Table 1:** Clinicopathological characteristics.

Variable	N = 289
**Age (years)**	
Median (range)	63 (36–82)
	**N (%)**
**Gender**	
Female	12 (4.2)
Male	277(95.8)
**Alcohol abuse (glasses per day)**	
No	71 (24.6)
Mild (1)	89 (30.8)
Moderate (2–4)	78 (27.0)
Heavy (>5)	51 (17.6)
**Smoking status (packet-years)**	
Never/Ex smoker	14 (4.8)/20 (6.9)
1–10	3 (1.0)
11–20	40 (13.8)
21–30	40 (13.8)
>30	172 (59.5)
**Localization**	
Glottic	125 (43.3)
Subglottic	6 (2.1)
Supraglottic	135 (46.7)
Transglottic	23 (8.0)
**Histological grade**	
1	107 (37.0)
2	121 (41.9)
3	41 (14.2)
Missing data	20 (6.9)
**T stage**	
T1/T2	21 (7.3)/43 (14.9)
T3/T4	140 (48.4)/85 (29.4)
**N stage (clinical/radiological)**	
N0	243 (84.1)
N1	21 (7.3)
N2	24 (8.3)
Missing data	1 (0.3)
**Surgery**	
Chordectomy	19 (6.6)
Total laryngectomy	243 (84.1)
Other surgical procedures	27(9.3)
**Neck dissection**	
Yes/No	84 (29.1)/205 (70.9)
**Postoperative irradiation**	
Yes/No	93 (32.2)/196 (67.8)

At a median follow-up time of 74.5 months, 135 (46.7%) patients had relapsed and 117 (40.5%) had died. The median and 5-year DFS were 94.5 months (95% CI: 80.9–108 months) and 60.8%, respectively, while the median and 5-year OS were 106.3 months (95% CI: 89.3–123.3 months) and 66.1%, respectively. As expected, median OS was significantly shorter for patients with lymph-node positive disease as compared to those with lymph-node negative (28.7 vs 106.5 months, p<0.001), for patients older than 63 years of age at diagnosis as compared to younger ones (87.3 vs 139.1 months, p = 0.006) and for patients whose tumors had a subglottic or transglottic location as compared to those whose tumors were located supraglottically (82.8 vs 117.5 months, p = 0.01). As compared to patients who had initially undergone total laryngectomy, patients who had undergone more conservative surgery experienced statistically significantly shorter median DFS (55.9 vs 106.2 months, p = 0.011) but not OS (124.8 vs 106.3 months, p = 0.671), probably due to the effect of salvage laryngectomy.

### IHC Expression of IGFR and correlation with outcome

**Figure 2:**
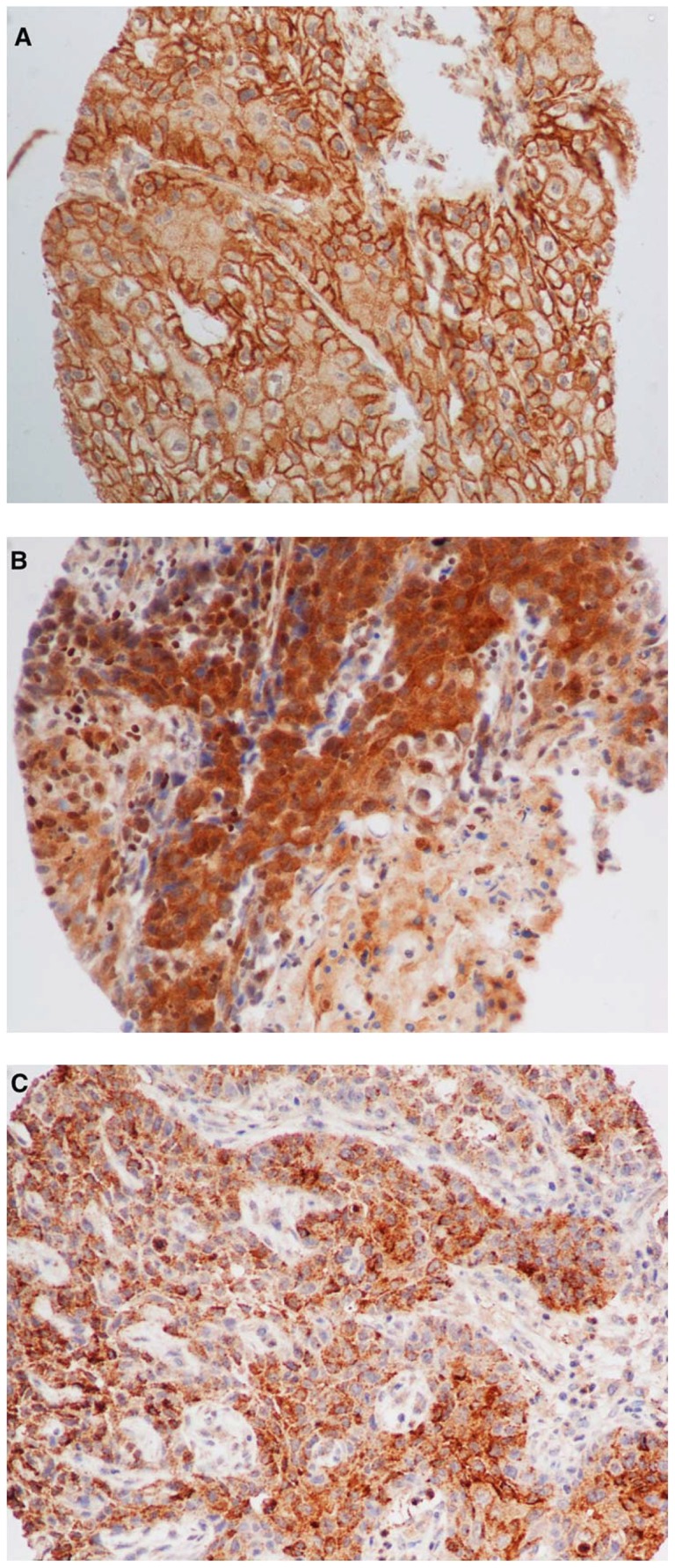
Illustrative examples of immunohistochemical expression and immunoreactive score (IRS) evaluation on tissue-microarray cores. A. IGF1R-alpha strong membraneous staining in 100% of cancer cells (IRS = 9); B. IGF1R-beta strong cytoplasmic staining in 80% of cancer cells (IRS = 9); C. IGF2R strong cytoplasmic and membraneous staining in 80% of cancer cells (IRS = 9). All pictures: magnification X 40.

Results of IHC staining were obtained for 285 (98.6%) of tumor samples for all three markers (IGF1R-alpha, IGF1R-beta and IGF2R). The pattern of immunostaining for IGF1R-alpha was predominantly cytoplasmic and/or membranous and was moderate to strong (IRS≥3) in 56.1% of cases (Figure 2A). On the contrary, IGF1R-beta immunostaining was mostly cytoplasmic (Figure 2B). and was absent (IRS = 0) in 81.7% of cases, despite the existence of positive internal controls (macrophages and stroma). IGF2R immunostaining was predominantly cytoplasmic and was moderate to strong (IRS≥2) in 53.6% of cases, (Figure 2C). Of note, a significant co-expression was observed between IGF1R-alpha cytoplasmic and IGF1R-alpha membranous immunostaining (r = 0.28, p<0.0001) but not between IGF1R-alpha and IGF1R-beta (r = −0.02, p = 0.779), nor between IGF1R-alpha and IGF2R (r = 0.09, p = 0.133). These findings urged us to further explore the IGF1R-alpha cytoplasmic/membranous IHC expression in relation to clinicopathological variables.

**Figure 3:**
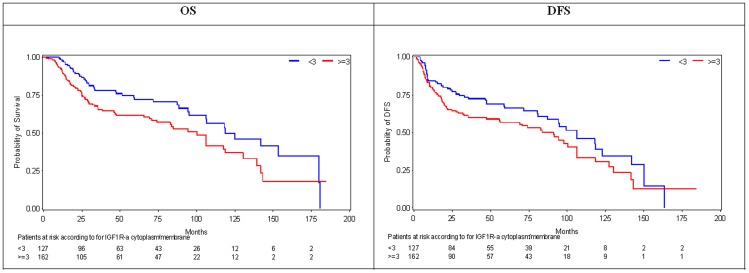
Kaplan- Meier Curves for overall survival (OS) and disease-free survival (DFS) according to IGF1R-alpha cytoplasm or membrane immunohistochemical expression (Median immunoreactive sore for IGF1R-alpha = 3).

As presented in Table 2, high expression of IGF1R-alpha (IRS≥3) was significantly associated with features of advanced disease, including TNM stage III or IV at diagnosis (p = 0.011) and total laryngectomy as the initial surgical procedure (p = 0.018). On the contrary, IGF1R-beta and IGF2R IHC expression was not associated with any of the clinicopathological variables studied (data not shown). In univariate analysis, patients whose tumors overexpressed cytoplasm/membrane IGF1R-alpha experienced marginally shorter median DFS as compared to those whose tumors did not (91.1 vs 106.2 months, p = 0.0538), and statistically significantly shorter median OS (100.3 vs 118.6 months, p = 0.0157), (Figure 3). None of the other IHC markers showed any association with survival outcomes, nor did the combination of IGF1R-alpha IHC expression with any other marker yield further information on patient outcome.

**Table 2:** Correlations between clinicopathological variables and IGF1R-alpha cytoplasmic/membraneous immunohistochemical expression.

		IGF1R-alpha cytoplasm or membrane IHC expression		
		<3	≥3	P-value
**Age (years)**	<55	35 (27.6)	37 (22.8)	0.655
	55–65	42 (33.1)	57 (35.2)	
	>65	50 (39.4)	68 (42.0)	
**Gender**	Female	5 (3.9)	7 (4.3)	0.871
	Male	122 (96.1)	155 (95.7)	
**Alcohol Abuse**	Yes	94 (74.0)	124 (76.5)	0.62
	No	33 (26.0)	38 (23.5)	
**Smoking Status (packet-years)**	1–30	34 (29.1)	38 (26.4)	0.572
	>30	67 (57.3)	91 (63.2)	
	No	16 (13.7)	15 (10.4)	
**Localization**	Glottic-Supraglottic	118 (92.9)	142 (87.7)	0.14
	Subglottic-Transglottic	9 (7.1)	20 (12.3)	
**T Stage**	I–II	37 (29.1)	27 (16.7)	**0.011**
	III–IV	90 (70.9)	135 (83.3)	
**N Stage**	N+	15 (11.9)	30 (18.5)	0.125
	N0	111 (88.1)	132 (81.5)	
**Surgery**	Other Type of Surgery	28 (22.0)	19 (11.7)	**0.018**
	Total Laryngectomy	99 (78.0)	143 (88.3)	
**Neck Dissection**	No	95 (74.8)	110 (67.9)	0.2
	Yes	32 (25.2)	52 (32.1)	
**Postoperative Irradiation**	No	86 (67.7)	110 (67.9)	0.973
	Yes	41 (32.3)	52 (32.1)	
**Histological Grade**	1	50 (39.4)	57 (35.2)	0.558
	2	50 (39.4)	71 (43.8)	
	3	16 (12.6)	25 (15.4)	
	Missing Data	11 (8.7)	9 (5.6)	
**Previous Tracheostomy**	No	105 (89.7)	117 (81.3)	0.056
	Yes	12 (10.3)	27 (18.8)	

### mRNA expression and correlation with outcome

Availability of tumor samples for assessment of mRNA expression for each one of the biomarkers is provided in Figure 1. In order to assess mRNA expression of the IGFR pathway-related genes and to screen their distribution for natural cut-offs, frequency histograms of RQ values for each biomarker were plotted and the corresponding boxplots were constructed (Figure S1). Receiver operator characteristic (ROC) analysis using the 3-year DFS as indicator and quartile analysis of distributions identified as cut-offs the median for IGF1R, IGFBP3, MAP2K1, MAPK9, PI3KR1 and SOCS2 and the 61^st^ percentile for PIK3CA. When markers' association were assessed in a continuous form, IGF1R mRNA expression was significantly associated with mRNA expression of IGFBP3 (r = 0.22, p = 0.0032), MAP2K1 (r = 0.50, p<0.0001), MAPK9 (r = 0.51, p<0.0001), PIK3CA (r = 0.34, p<0.0001), PIK3R1 (r = 0.37, p<0.0001) and SOCS2 (r = 0.27, p = 0.0014), indicating that IGF1R mRNA is co-expressed with all other important effectors of the IGF molecular pathway. The downstream molecules of the two main signaling cascades triggered by IGFR activation (MAPK9 with MAP2K1 and PIK3CA with PIK3R1) were also significantly correlated with each other in terms of mRNA expression. Finally, IGFBP3 mRNA expression was correlated with MAPK9, PIK3CA and SOCS2 mRNA expression (Table S1).

None of the biomarker's mRNA expression, assessed as continuous variable, was associated with any of the clinicopathological variables studied (data not shown). In univariate analysis and using the identified cut-off values, patients whose tumors had high MAPK9 mRNA expression experienced marginally longer median DFS (91.8 vs 81.0 months, p = 0.0665) and statistically significantly longer median OS (117.5 vs 87.3 months, p = 0.0344), as compared to patients whose tumors had low MAPK9 mRNA levels. Similarly, patients whose tumors had high PIK3CA mRNA expression experienced longer -albeit not significantly- median DFS (106.2 vs 80.5 months, p = 0.0723) and median OS (141.8 vs 94.5 months, p = 0.0726), as compared to patients whose tumors had low PIK3CA mRNA levels (Table S2).

### Correlation of IGF1R IHC expression with mRNA levels

We observed a non-significant trend for co-expression of IGF1R-alpha membranous or cytoplasmic staining and IGF1R-alpha mRNA levels, as evaluated with the Spearman's correlation test (r = 0.14, p = 0.0621), (Figure S2). However, there was no correlation of IGF1R-alpha IHC expression with mRNA levels of any of the other biomarkers evaluated (data not shown).

### Multivariate analysis

**Figure 4:**
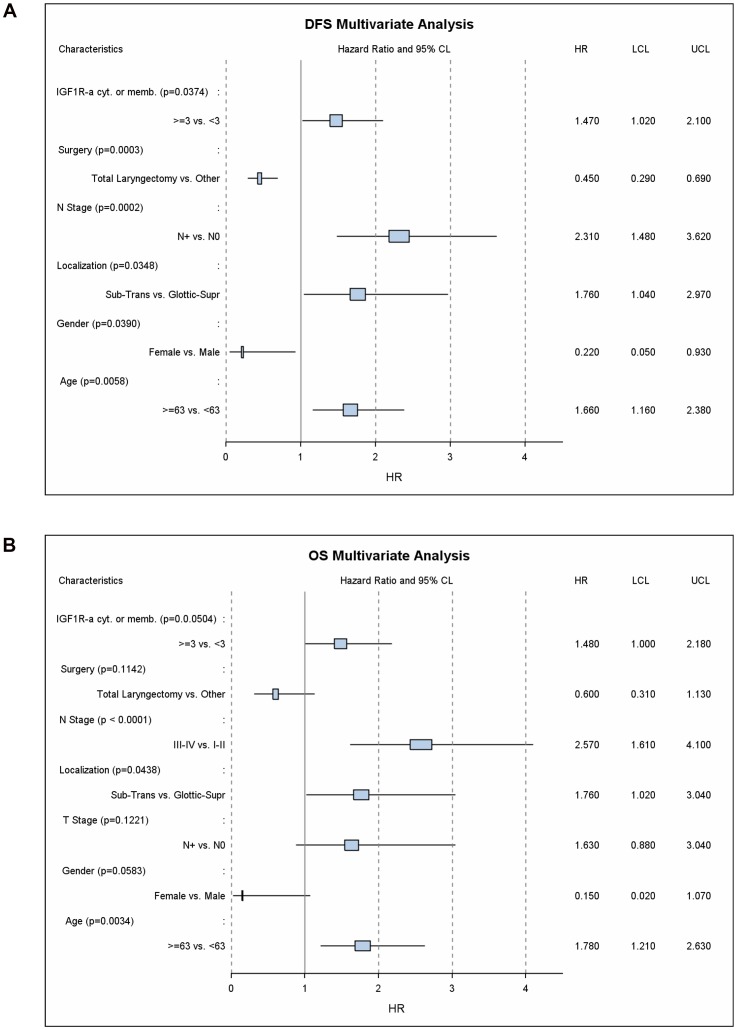
Forest plot illustration of the multivariate analysis of predictors for DFS (Panel A) and OS (Panel B) in operable laryngeal cancer.

In multivariate analysis, independent predictors for DFS were the type of surgery (laryngectomy vs conservative surgery), lymph node status (positive vs negative), sex (male vs female), age at diagnosis (above or below the median of 63 years), tumor location (subglottic/transglottic vs supraglottic/glottic) and IGF1R-alpha cytoplasmic or membranous IHC expression (high vs low), (Figure 4A). Potent predictors for DSF were male sex, which was associated with a more than four-fold increase in the risk for relapse (HR = 4.459, 95%CI 1.079-18.436, p = 0.039) and the presence of infiltrated (positive) lymph nodes, which was associated with a more than two-fold increase in the same risk (HR = 2.313, 95%CI 1.477–3.622, p = 0.0002). Importantly, patients whose tumors overexpressed IGF1R-alpha had a 46.6% increase in the risk for relapse, after adjustment for all the above-mentioned predictive factors (HR = 1,466, 95% CI: 1.022–2.102, p = 0.0374).

**Figure 5:**
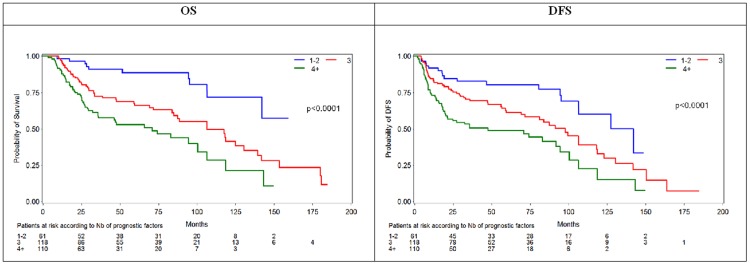
Kaplan- Meier Curves for disease-free survival (DFS) and overall survival (OS) according to the number of negative prognostic factors from the multivariate analysis (blue line: 1–2 factors; red line: 3 factors; green line: 4 or more factors).

The final model for OS included the same clinicopathological variables plus T-stage (T3/T4 vs T1/T2). The most powerful adverse prognostic factors for OS were infiltrated lymph nodes (HR = 2.569, 95%CI: 1.610–4.100, p<0.0001), age more than 63 years (HR = 1.785, 95%CI: 1.211–2.630, p = 0.0034) and subglottic/transglottic location (HR = 1.756, 95%CI: 1.016–3.036, p = 0.0438). As shown in Figure 4B, increased cytoplasmic or membranous IGF1R-alpha protein expression, was associated with an absolute 47.5% increase in the risk of death after adjustment for all other clinicopathological parameters and this result was marginally significant (HR = 1.475, 95%CI: 1.000–2.178, p = 0.0504). Notably, the incorporation of IGF1R IHC expression in the multivariate model enabled the stratification of patients in three groups with distinct prognosis depending on the number of adverse prognostic factors (1–2, 3 and 4 or more adverse factors for the favorable, intermediate and unfavorable prognosis group respectively). For example, median OS was 70.7 months (95%CI: 35.4–100.3 months) for patients in the unfavorable prognosis group and 106.3 months (95%CI: 84.8–130.4 months) in the intermediate prognosis group, whereas it was not reached in the favorable prognosis group (Figure 5).

## Discussion

In the present study, the only one to our knowledge exploring the prognostic role of IGF1R in early laryngeal cancer, we have shown that increased IGF1R-alpha cytoplasmic and/or membraneous expression, as assessed by immunohistochemistry and quantified with the IRS system, is an independent adverse prognostic factor for recurrence and survival in patients with early (surgically resected) squamous-cell carcinoma of the larynx. IGF1R-alpha expression remained a significant prognostic factor for both DFS and OS even after adjustment for well-defined clinicopathological variables, such as cervical lymph node involvement, age and tumor location. These results are in line with recently published important evidence on gene arrays [22] that highlight the importance of the IGFR-mediated molecular pathway in laryngeal carcinogenesis and progression; Of note, current treatment modalities in early stages of laryngeal cancer include surgical resection with or without adjuvant radiotherapy, as used in our cohort, rendering thus our results timely and clinically relevant. Moreover, the feasibility and reproducibility of the IRS evaluation in independent pathology laboratories render IGF1R-alpha protein expression an appealing biomarker for routine clinical practice that may serve as a decision tool for more aggressive treatment in early laryngeal cancer.

The IGFR-mediated molecular pathway has consistently been implicated in neoplastic transformation and progression in a number of human malignancies, including those of the aerodigestive tract and has thus long attracted attention both as a potential prognostic biomarker [9], [10] and as a potential target for therapeutic intervention [11], [12]. Data regarding IGFR prognostic value in NSCLC are rather conflicting [3], [4], [29], although a recent study [5] showed that IGF1R protein expression is higher in squamous-cell histologies and concluded that IGF1R protein and gene expression were not associated with survival, whereas *IGF1R* gene copy number harbored prognostic value. In laryngeal cancer, the paucity of data does not allow for safe conclusions to be drawn: IGF1R and IGFBP3 serum levels were not identified as significant predictors of clinical outcome in the only large cohort of 540 patients with SCCHN, including 440 patients with laryngeal cancer, published to date [9]; This study, however, assessed exclusively serum levels of IGF1R and IGFBP3 and not tumoral mRNA levels or IHC expression, as in our cohort. Interestingly, IGFBP3 has been reported to act as a suppressor of vascular endothelial growth factor (VEGF) in SCCHN angiogenesis [16] and to be downregulated in the early phases of head and neck carcinogenesis [30]. In oral squamous-cell carcinoma, IGFBP3 mRNA expression has been correlated with a more favorable outcome, further supporting its role as an IGF1 inactivator [31]. In a recently published study [32], the combination of IGF1R and IGFBP3 IHC overexpression was prognostic for poor survival in a cohort of 131 patients with SCCHN. In our cohort, consisted exclusively of patients with laryngeal cancer, the incorporation of IGFBP3 IHC expression did not improve the prognostic capacity of IGF1R alone. Finally, the role of IGF2R in laryngeal cancer remains to be elucidated [33], although it appears probable that the lack of a tyrosine kinase domain deprives the receptor from the ability to transduce proliferative signals and may thus explain the absence of prognostic value of the marker observed in our cohort.

An important element in the design of our study was the use of separate monoclonal antibodies targeting the two distinct isoforms of the IGF1R protein (alpha and beta). This discrimination is of particular importance since only IGF1R-alpha is able to bind with IGF1 and IGF2 and is thus critical for IGFR-mediated signal transduction [11]. Therefore, antibodies not aiming specifically at the alpha epitope may be inappropriate for analysis and their use may render results uninterpretable. In our cohort, the addition of IGF1R-beta IHC expression did not improve prognostic capacity of IGF1R-alpha alone, suggesting a distinct biological role for the former.

As mentioned in the literature [10], activation of IGF1R triggers the activation of both the MAPK and the PI3K cytoplasmic effector pathways: the activation of these pathways was also observed in our study by the increased expression of mRNA levels of important elements for both pathways (MAPK9, MAP2K1, PIK3CA and PIK3R1 but not SOCS2). Of note, increased mRNA levels of MAPK9, in particular, were associated with a more favorable clinical outcome; this paradox may be explained by recent evidence suggesting that the specific member of the MAPK family, and especially in the c-Jun N-terminal kinase 2 (JNK2) isoform, may act as a negative regulator of cellular proliferation [11]. Nevertheless, there was no significant association between IGF1R IHC expression and IGF1R mRNA levels (p = 0.0621), neither did gene expression of any of the markers bear prognostic value for clinical outcomes of interest. Possible explanations for the discordance between gene and protein expression include failure of mRNA to translate into the specific protein of interest due to cleavage, translational silencing or alternative splicing and mRNA degradation in the paraffin-embedded tumor block during fixation. Given that IGF1R isoforms are produced mainly by protein cleavage [10], the levels of IGF1R-alpha and IGF1R-beta are not expected to be associated with IGF1R mRNA levels. Our results suggest that IGF1R protein isoforms, as detected by IHC, are more sensitive and biologically relevant markers for the study of the IGFR-mediated pathway compared to IGF1R mRNA levels. Potential limitations of immunohistochemistry, as performed in our study, include the subjectivity of IRS evaluation due to inter-observer variability, lack of antibody specificity for a specific IGF1R isoform and IHC technique limitations due to the absence of standardized protocols for the use of IGF1R monoclonal antibodies outside research purposes.

In conclusion, we have found that IGF1R-alpha cytoplasmic/membranous overexpression, as assessed by IHC and evaluated with the IRS system, may serve as an independent adverse prognostic factor for recurrence and survival in patients with surgically resected squamous-cell carcinoma of the larynx. Further studies should prospectively validate the prognostic role of IGF1R-alpha in early laryngeal cancer and its ability to identify subjects at high risk for relapse who may necessitate a more aggressive treatment. Future studies should also investigate the value of the marker in predicting response and outcome in patients receiving anti-IGF1R agents.

## Supporting Information

Table S1 Correlations of mRNA levels among biomarkers of the IGFR pathway.
(DOC)

Table S2 Univariate analysis of mRNA level correlation with DFS and OS for each biomarker of the IGFR pathway. Cutoffs at 50^th^ percentile unless otherwise indicated.
(DOC)

Figure S1 Histograms and corresponding boxplots of mRNA levels for each biomarker.
(TIF)

Figure S2 Co-expression of IGF1R-alpha membraneous or cytoplasmic staining and IGF1R-alpha mRNA levels, as evaluated with the Spearman's correlation test.
(TIF)
