# Supplementary material for: Insulin-Like Growth Factor 1 Receptor (IGF1R) Expression and Survival in Operable Squamous-Cell Laryngeal Cancer
Source: PLoS One. 2013 Jan 24;8(1):e54048. doi: 10.1371/journal.pone.0054048 (PMC3554755; doi:10.1371/journal.pone.0054048)
Supplement: Table S1 — Correlations of mRNA levels among biomarkers of the IGFR pathway. (DOC) [file pone.0054048.s001.doc]

**Supplementary Table 1:**  Correlations of mRNA levels among biomarkers of the IGFR pathway.

| **Variable** | **With Variable** | **N** | **Sample Correlation** | **95%CI** | | **p Value** |
| --- | --- | --- | --- | --- | --- | --- |
|  |  |  |  | **LL** | **UL** |  |
| IGF1R | IGFBP3 | 174 | **0.22** | 0.08 | 0.36 | 0.0032 |
|  | MAP2K1 | 171 | **0.50** | 0.37 | 0.60 | <.0001 |
|  | MAPK9 | 148 | **0.51** | 0.38 | 0.62 | <.0001 |
|  | PIK3CA | 158 | **0.34** | 0.20 | 0.47 | <.0001 |
|  | PIK3R1 | 164 | **0.37** | 0.23 | 0.50 | <.0001 |
|  | SOCS2 | 133 | **0.27** | 0.11 | 0.42 | 0.0014 |
| IGFBP3 | MAP2K1 | 170 | **0.03** | -0.12 | 0.18 | 0.6758 |
|  | MAPK9 | 149 | **0.21** | 0.05 | 0.36 | 0.0097 |
|  | PIK3CA | 158 | **0.16** | 0.00 | 0.31 | 0.0460 |
|  | PIK3R1 | 166 | **0.14** | -0.01 | 0.29 | 0.0673 |
|  | SOCS2 | 134 | **0.19** | 0.02 | 0.35 | 0.0279 |
| MAP2K1 | MAPK9 | 148 | **0.50** | 0.37 | 0.61 | <.0001 |
|  | PIK3CA | 157 | **0.26** | 0.11 | 0.40 | 0.0010 |
|  | PIK3R1 | 163 | **0.31** | 0.16 | 0.44 | <.0001 |
|  | SOCS2 | 132 | **0.12** | -0.05 | 0.28 | 0.1745 |
| MAPK9 | PIK3CA | 138 | **0.33** | 0.17 | 0.47 | <.0001 |
|  | PIK3R1 | 146 | **0.52** | 0.39 | 0.63 | <.0001 |
|  | SOCS2 | 120 | **0.11** | -0.07 | 0.28 | 0.2275 |
| PIK3CA | PIK3R1 | 153 | **0.44** | 0.30 | 0.56 | <.0001 |
|  | SOCS2 | 126 | **0.21** | 0.04 | 0.37 | 0.0165 |
| PIK3R1 | SOCS2 | 130 | **0.33** | 0.17 | 0.48 | <.0001 |
