# Supplementary material for: Insulin-Like Growth Factor 1 Receptor (IGF1R) Expression and Survival in Operable Squamous-Cell Laryngeal Cancer
Source: PLoS One. 2013 Jan 24;8(1):e54048. doi: 10.1371/journal.pone.0054048 (PMC3554755; doi:10.1371/journal.pone.0054048)
Supplement: Table S2 — Univariate analysis of mRNA level correlation with DFS and OS for each biomarker of the IGFR pathway. Cutoffs at 50th percentile unless otherwise indicated. (DOC) [file pone.0054048.s002.doc]

**Supplementary Table 2:**  Univariate analysis of mRNA level correlation with DFS and OS for each biomarker of the IGFR pathway. Cutoffs at 50^th^ percentile unless otherwise indicated.

OS

|  | | | | | **Range** | |  | **95% CI** | |  |
| --- | --- | --- | --- | --- | --- | --- | --- | --- | --- | --- |
|  | | **N** | **Events** | **%censored** | **Min** | **Max** | **Median** | **Lower** | **Upper** | **P-value** |
| IGF1R | High | 87 | 39 | 55.2 | 1.5 | 181.2 | 106.3 | 47.5 | 141.8 | 0.5646 |
|  | Low | 88 | 32 | 63.6 | 5.8 | 153.3 | 100.3 | 69.1 | 153.3 |  |
| IGFBP3 | High | 88 | 36 | 59.1 | 7.4 | 181.2 | 100.3 | 51.3 | . | 0.9780 |
|  | Low | 88 | 35 | 60.2 | 1.5 | 154.8 | 106.2 | 83.8 | 124.8 |  |
| MAP2K1 | High | 86 | 33 | 61.6 | 5.8 | 181.2 | 117.5 | 87.3 | . | 0.0931 |
|  | Low | 87 | 36 | 58.6 | 1.5 | 141.8 | 69.1 | 35.4 | 141.8 |  |
| MAPK9 | High | 76 | 28 | 63.2 | 5.8 | 181.2 | 117.5 | 84.8 | . | **0.0344** |
|  | Low | 77 | 35 | 54.5 | 1.5 | 130.4 | 87.3 | 35.4 | 124.8 |  |
| PIK3CA | High | 81 | 31 | 61.7 | 1.5 | 181.2 | 106.2 | 83.8 | . | 0.4784 |
|  | Low | 81 | 35 | 56.8 | 7.4 | 154.8 | 94.5 | 47.5 | 124.8 |  |
| PIK3CA (61^st^ percentile) | High | 63 | 21 | 66.7 | 7.4 | 181.2 | 141.8 | 87.3 | . | 0.0726 |
|  | Low | 99 | 45 | 54.5 | 1.5 | 154.8 | 94.5 | 47.5 | 117.5 |  |
| PIK3R1 | High | 84 | 36 | 57.1 | 1.5 | 181.2 | 106.2 | 65.8 | 153.3 | 0.4469 |
|  | Low | 84 | 30 | 64.3 | 5.8 | 141.8 | 117.5 | 59.3 | 141.8 |  |
| SOCS2 | High | 69 | 29 | 58.0 | 7.4 | 154.8 | 141.8 | 29.9 | . | 0.9909 |
|  | Low | 69 | 28 | 59.4 | 5.8 | 153.3 | 100.3 | 46.3 | 153.3 |  |

**DFS**

|  | | | | | **Range** | |  | **95% CI** | |  |
| --- | --- | --- | --- | --- | --- | --- | --- | --- | --- | --- |
|  | | **N** | **Events** | **%censored** | **Min** | **Max** | **Median** | **Lower** | **Upper** | **P-value** |
| All patients |  | 289 | 135 | 53.3 | 0.4 | 184.6 | 94.5 | 81.0 | 106.3 | . |
| IGF1R | High | 87 | 44 | 49.4 | 0.4 | 142.4 | 83.8 | 47.5 | 127.4 | 0.8153 |
|  | Low | 88 | 38 | 56.8 | 1.9 | 122.9 | 87.3 | 35.4 | 122.9 |  |
| IGFBP3 | High | 88 | 41 | 53.4 | 0.4 | 142.4 | 100.3 | 33.0 | 127.4 | 0.9035 |
|  | Low | 88 | 41 | 53.4 | 1.5 | 141.8 | 87.3 | 47.5 | 106.2 |  |
| MAP2K1 | High | 86 | 40 | 53.5 | 1.9 | 142.4 | 91.1 | 56.5 | 127.4 | 0.3384 |
|  | Low | 87 | 40 | 54.0 | 0.4 | 141.8 | 69.1 | 24.8 | 141.8 |  |
| MAPK9 | High | 76 | 33 | 56.6 | 1.9 | 142.4 | 91.8 | 69.1 | 127.4 | 0.0665 |
|  | Low | 77 | 39 | 49.4 | 0.4 | 130.4 | 81.0 | 22.2 | 106.2 |  |
| PIK3CA | High | 81 | 38 | 53.1 | 1.5 | 142.4 | 91.8 | 46.8 | 127.4 | 0.4100 |
|  | Low | 81 | 39 | 51.9 | 0.4 | 130.4 | 80.5 | 47.5 | 100.3 |  |
| PIK3CA (61^st^ percentile) | High | 63 | 27 | 57.1 | 2.9 | 142.4 | 106.2 | 56.5 | 141.8 | 0.0723 |
|  | Low | 99 | 50 | 49.5 | 0.4 | 130.4 | 80.5 | 35.4 | 100.3 |  |
| PIK3R1 | High | 84 | 40 | 52.4 | 1.5 | 142.4 | 83.8 | 56.5 | 106.3 | 0.5007 |
|  | Low | 84 | 37 | 56.0 | 1.9 | 141.8 | 91.8 | 46.8 | 141.8 |  |
| SOCS2 | High | 69 | 32 | 53.6 | 2.9 | 142.4 | 87.3 | 22.0 | . | 0.7317 |
|  | Low | 69 | 33 | 52.2 | 0.4 | 128.6 | 69.1 | 32.5 | 100.3 |  |
