# Supplementary figures and images for: Insulin-Like Growth Factor 1 Receptor (IGF1R) Expression and Survival in Operable Squamous-Cell Laryngeal Cancer
Source: PLoS One. 2013 Jan 24;8(1):e54048. doi: 10.1371/journal.pone.0054048 (PMC3554755; doi:10.1371/journal.pone.0054048)

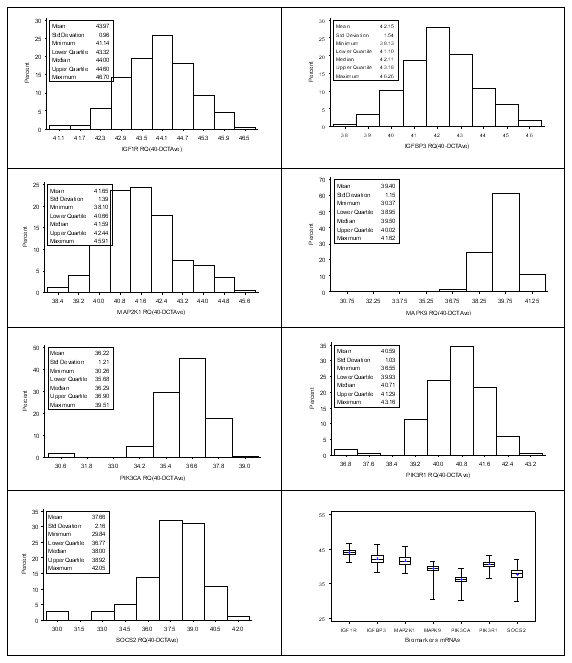

Supplement: Figure S1 — Histograms and corresponding boxplots of mRNA levels for each biomarker. (TIF) [file pone.0054048.s003.tif]
